# Medical students can teach communication skills – a mixed methods study of cross-year peer tutoring

**DOI:** 10.1186/s12909-017-0939-7

**Published:** 2017-06-15

**Authors:** Osamu Nomura, Hirotaka Onishi, Hiroyuki Kato

**Affiliations:** 1 Department of Integrated Medical Education, Graduate School of Medicine, Hirosaki University, 1 Zaifu-cho, Hirosaki city, Aomori, Japan; 2 Division of Pediatric Emergency Medicine, Tokyo Metropolitan Children’s Hospital, 2-8-29 Musashidai, Fuchu city, Tokyo Japan; 3 International Research Center for Medical Education, Graduate School of Medicine, The University of Tokyo, 7-3-1 Hongo, Bunkyo-ku, Tokyo, Japan

**Keywords:** Cross-year peer tutoring, Peer assisted learning, Communication training, Mixed methods study, Non-inferior trial

## Abstract

**Background:**

Cross-year peer tutoring (CYPT) of medical students is recognized as an effective learning tool. The aim of this study is to investigate the non-inferiority of the objective outcome of medical interview training with CYPT compared with the results of faculty-led training (FLT), and to explore qualitatively the educational benefits of CYPT.

**Methods:**

We conducted a convergent mixed methods study including a randomized controlled non-inferiority trial and two focus groups. For the CYPT group, teaching was led by six student tutors from year 5. In the FLT group, students were taught by six physicians. Focus groups for student learners (four tutees) and student teachers (six tutors) were conducted following the training session.

**Results:**

One hundred sixteen students agreed to participate. The OSCE scores of the CYPT group and FLT group were 91.4 and 91.2, respectively. The difference in the mean score was 0.2 with a 95% CI of −1.8 to 2.2 within the predetermined non-inferiority margin of 3.0. By analyzing the focus groups, we extracted 13 subordinate concepts and formed three categories including ‘Benefits of CYPT’, ‘Reflections of tutees and tutors’ and ‘Comparison with faculty’, which affected the interactions among tutees, tutors, and faculty.

**Conclusions:**

CYPT is effective for teaching communication skills to medical students and for enhancing reflective learning among both tutors and tutees.

## Background

Cross-year peer tutoring (CYPT) among medical students has come to be recognized as an effective learning tool and its benefits have been well documented in a range of educational settings for healthcare providers in recent years [1–4]. Medical schools are starting to recognize the importance of CYPT and almost half of US and UK medical schools now offer some kind of CYPT program [5, 6].

Research suggests that CYPT would be effective in teaching both basic and specialized content if the teaching framework is well-structured [7]. On the basis of such findings, numerous studies have been conducted to test the efficacy of clinical teaching activities dealing with physical examinations, basic surgical procedures, and cardiopulmonary resuscitation [8–10], and found that the objective learning outcomes of students taught using CYPT were equivalent to those of students taught using faculty led training (FLT) [11]. In terms of medical interviews or communication training, a few randomized controlled trials on CYPT have been conducted, but these failed to show the superiority of the objective learning outcome in comparison with the FLT [12, 13].

Many reports have examined the efficacy of CYPT in undergraduate medical education, comparing the objective outcome of CYPT to that of FLT [7, 12–16], and found no statistically significant difference between the two teaching methods, *suggesting* that both were equally efficacious. By the same token, equivalence or non-inferiority between the two methods could not be established due to the fact that there was no significant statistical difference in the educational outcome between CYPT and FLT in the superiority design [17]. To prove the hypothesis that student-led teaching was not inferior to faculty-led teaching, a non-inferiority study, which is now increasingly recognized as an effective methodological tool for professional healthcare education research [18–20], was required. In this study protocol, non-inferiority occurred when the lower limit of the 95% confidence interval (CI) for the difference in educational outcomes between the two teaching methods did not exceed the non-inferiority margin. The non-inferiority margin, which expresses the minimum difference regarded to be educationally (or clinically) meaningful [21], should be defined in the process of designing the study.

Researchers have also been interested in why CYPT is beneficial to medical education. Cognitive congruence between student learners (tutee) and student teachers (tutors) is now recognized as the key concept behind the efficacy of CYPT [3, 22]. However, only a few qualitative studies have investigated the factors contributing to the efficacy of CYPT in the context of undergraduate communication training [23].

We hypothesized that the objective educational outcomes of CYPT would be non-inferior to those of FLT, and that there would be subjective educational benefits for both tutees and tutors in CYPT.

## Methods

### Objectives

The aim of this study was to confirm the non-inferiority of the objective educational outcomes of medical interview training using CYPT compared with faculty-led training (FLT), and to explore qualitatively the educational benefits of CYPT for undergraduate communication training.

### Program structure

Hirosaki University in Hirosaki, Japan provides a six-year undergraduate medical education program. CYPT sessions are conducted in the medical interview training module in the pre-clinical intensive training course for fourth-year medical students, who have no experience in clinical training. This pre-clinical training includes a 4-week off-the-job course consisting of training in medical interviews, systematic physical examination, basic surgical skills, basic cardiopulmonary resuscitation, and a lecture series on professionalism. Students are required to take a summative Objective Structured Clinical Examination (OSCE) covering the basic clinical skills after completion of the course. OSCE is managed by the government-run Common Achievement Test Organization (CATO) in Japan, and includes assessments of skills in medical interviewing, systematic physical examination, basic surgery, and basic cardiopulmonary resuscitation.

In the medical interview training module, students take four sessions consisting of a lecture on basic interviewing skills (1.5 h), a large-group demonstration of a medical interview session (3 h), a structured small-group role-play of a medical interview (3 h), and a similar small group medical interview session with actors playing the role of patients (3 h).

### Study setting

The present study was conducted during the third small-group structured role-play session (3 h) out of four sessions in the medical interview training module.

### Tutor selection and tutor training

Our student tutors were recruited on a voluntary basis from among the ranks of fifth year students without any financial incentive. Although all the tutors attended the same session as students in the previous year, none of them had any experience as a tutor and/or teacher at the university. As this trial was conducted using communication training within the standard medical curriculum, all six student tutors were involved in the training of the CYPT intervention group. Under the supervision of faculty physicians, student tutors received one hour of tutor-training in terms of providing effective feedback in the medical interview training sessions as described in the previous studies [1, 24].

### Study design

As part of this convergent mixed methods study, we conducted a randomized controlled non-inferiority trial for the quantitative component concurrently with two focus groups for the qualitative component, and analyzed the results of both after completion. This study was performed from January 2014 to February 2014.

### Quantitative study

**Fig. 1:**
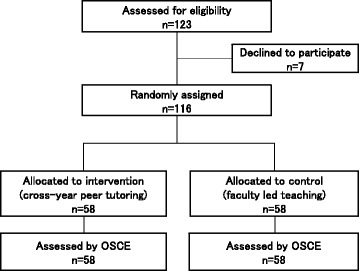
Trial profile

The randomized non-inferiority trial was performed using fourth-year students randomly assigned to two groups. Of the 123 4th-year students, 116 students agreed to participate. We randomly assigned 58 students to the CYPT group and 58 to the FLT group by computer-generated, permuted block randomization (Fig. 1). For the CYPT group, teaching was led by a group of six student tutors from year 5. In the FLT group, students were taught by a group of six physicians. Seven students who declined to participate were removed from the study cohort and returned to the normal courses taught by the faculty.

Efficacy was determined by comparing the performance of students in the CYPT group with that of students in the FLT group on a ten-minute OSCE for the medical interview. The OSCE was conducted one week after the training session under the supervision of CATO.

### Statistical analysis

The quantitative part of this study was based on the hypothesis that CYPT would not be inferior to FLT in relation to the OSCE score (0 to 100). We defined the non-inferiority margin as a score of 3.0 considered to be educationally meaningful in our setting and context. This decision was made after careful discussion among the researchers based on the previous test results and their experiences at our university.

We calculated that a sample size of 114 students would give a power of 80%, sufficient to determine whether CYPT was not inferior to FLT in relation to the OSCE score while also taking into account the non-inferiority margin (3.0), a one-sided α error of 0.025, and a standard deviation estimate of 5.7 based on the OSCE scores obtained in the previous year at our university.

### Qualitative study

Two focus group attended by the four tutees from the CYPT group and all six tutors were held separately prior to the OSCE. The four tutees volunteered to join the focus group in response to our invitation, which was announced to all 4th-year-students at the beginning of the medical interview training module. Discussion was structured by a series of questions, but participants were encouraged to comment freely on any aspect of this experience. They were interviewed by the first author in a private room at the university hospital for approximately 60 min. The interview was audiotaped and transcribed verbatim. A Modified Grounded Theory Approach, a version of the grounded theory approach adapted for greater practicability, was used for analysis [25]. First, we reviewed the transcripts to identify the concepts and compared the concepts with each other until no further new concepts emerged. Next, we analyzed the relationships among the concepts, and generated categories. Finally, we reviewed the relationships between the categories while referring to theoretical notes and made diagrams of the concepts and categories showing their interactions. Through the process of interpretive analysis, we focused on sample characteristics and repeatedly reviewed the data. To enhance the credibility of the analysis, the first author mainly performed the analysis and discussed the results periodically with the second author. All discrepancies were discussed until agreement was achieved. The third author afterwards reviewed the conceptual model for triangulation.

### Ethical considerations

This study was approved by the ethics committee of Hirosaki University Graduate School of Medicine and written informed consent was obtained from all participants.

## Results

### Quantitative results

The proportion of male students and students who had obtained their bachelor’s degree before entering medical school was similar between the two groups.

**Fig. 2:**
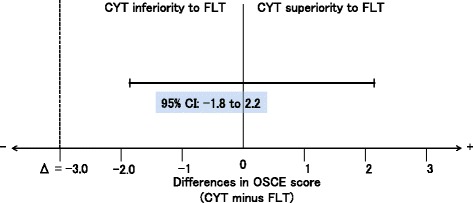
Non-inferiority analysis. Error bars indicate two-sided 95% CI of the difference in the OSCE score between the CYPT and FLT groups. The broken line delineating the difference in the score (= 3.0) shows the non-inferiority margin; the region to the right of the margin indicates the zone of inferiority. The lower limit of the CI lies to the right of the non-inferiority margin, demonstrating non-inferiority of CYPT relative to FLT

The OSCE scores of the CYPT group and FLT group were 91.4 and 91.2, respectively (Table 1). The difference in the mean scores was 0.2 with a 95% CI of −1.8 to 2.2, which did not exceed the predetermined non-inferiority margin of 3.0 (Fig. 2).

**Table 1:** Participant characteristics and OSCE score

	CYPT group (*n* = 58)	FLT group (*n* = 58)
Male, n (%)	43 (74.1)	45 (77.6)
Bachelor, n (%)	9 (15.5)	9 (15.5)
OSCE score (SD)	91.4 (5.5)	91.2 (5.4)

Note. *CYPT* cross-year tutoring; *FLT* faculty led training; *OSCE* objective structured clinical evaluation; *SD* standard deviation

### Qualitative results

**Fig. 3:**
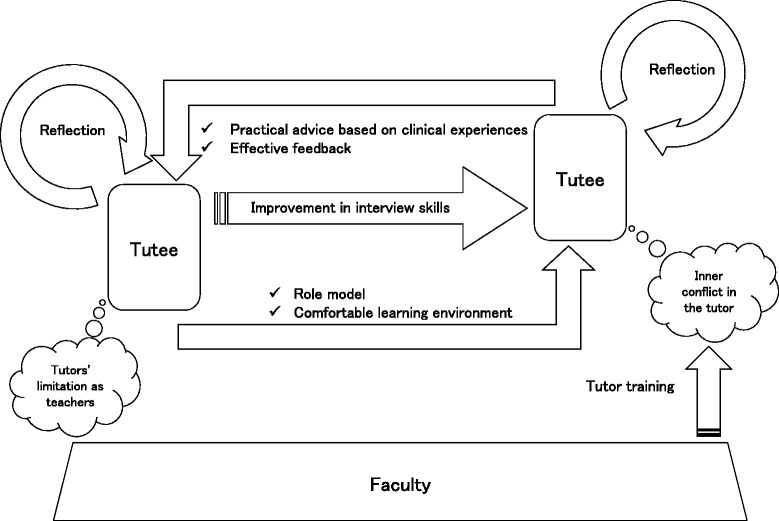
Overview of interaction on cross-year peer tutoring. Interaction among tutees, tutors and faculty in cross-year peer tutoring

We extracted 13 subordinate concepts and finally formed three categories of factors affecting interactions among tutees, tutors, and faculty in CYPT as shown below (Table 2 and Fig. 3):

- Benefits of CYPT
- Reflections of tutees and tutors
- Comparison with faculty

- Benefits of CYPT

**Table 2:** List of categories and concepts

Category	Concept	Example of feedback
Benefits of CYPT	Role Model	*By following the tutors’ example, we could understand what we needed to do. (tutee)*
	Confortable Learning Environment	*They were comfortable enough to give us kind advice from our perspective as students. (tutee)*
	Effective Feedback	*They gave us a lot of valuable feedback about what we did, how we performed, and how we could improve. (tutee)*
	Practical Advice Based on Clinical Experiences	*I was impressed by how they taught us by sharing their experience of bedside training with us. (tutee)*
	Improvement of Interview Skills	*Students interviewed in the latter part of the session improved their skills by accepting our advice based on the experiences of the students interviewed in the earlier part. It’s like learning from other people’s mistakes. (tutor)*
Reflections of tutees	Differences in Competency Between Tutors and Tutees	*We can reflect on what we need to learn this year by comparing our own experiences with those of the tutors. (tutee)*
	Prediction of Tutors’ Reflection	*I guess that tutors can become confident by comparing themselves with the junior students; by fielding our questions, they may see what they need to improve in their own knowledge. (tutee)*
Reflections of tutors	Developing Self-confidence	*I developed confidence through the experience of teaching junior students.(tutor)*
	Teaching as Another Opportunity for Learning	*I felt tutoring to be a second opportunity for me to learn. (tutor)*
	Reflecting on Ones’ Attitude as a Learner	*During this teaching experience, I reflected on what my attitude was like when I was being taught and the fact that I need to be more active in studying. (tutor)*
Comparison with faculty	Tutors’ Limitation as Teachers	*I felt that faculty teachers might be better than student tutors in teaching the ideal style and language of medical interviews. (tutee)*
	Inner Conflict in the Tutor	*Before the session I was nervous about whether I would be able to teach junior students. (tutor)*
		*I felt that we had an advantage over the faculty in teaching in this session because we took it just last year, and understood what tutees wanted to know. (tutor)*
	Tutor Training is Essential	*We would have been in trouble if we hadn’t had any teacher training. (tutor)*

The tutees recognized the tutors as role models (Role Model) and felt that tutors offered a comfortable learning environment (Comfortable Learning Environment). They also felt that the tutors provided effective feedback and practical advice based on their own experiences in clinical practice (Effective Feedback & Practical Advises Based on Clinical Experience).

Tutors recognized improvement in tutees’ interview skills. (Improvement in Interview Skills)

b) Reflections of tutees and tutors

Tutees reflected on the differences in competency between tutors and tutees (Differences in Competency Between Tutors and Tutees). Moreover, tutees reported that they thought it likely that tutors would be more inclined to reflect on their own role after their experience in CYPT (Prediction of Tutors’ Reflection).

Conversely, the tutors reported that they felt more self-confident and acquired deeper understanding of content by teaching (Development of Self-confidence & Teaching as Another Opportunity for Learning). In addition, they reflected on their usual attitude when being taught by the faculty (Reflection on Ones’ Attitude as a Learner).

c) Comparison with faculty

Tutees reported that tutors experienced difficulty on a few occasions while teaching because of insufficient experience (Tutors’ Limitation as Teachers). While tutors recognized that they might have an advantage over the faculty when teaching junior students, they also felt anxious before teaching (Inner Conflict in the Tutor). The tutors stated that tutor training by the faculty was essential to resolving this anxiety (Tutor Training is Essential).

## Discussion

This is the first mixed methods study including a non-inferiority trial investigating the efficacy of cross-year tutoring, to the best of our knowledge. We showed the non-inferiority of CYPT against FLT for medical interview training in pre-clinical medical students. Furthermore, our qualitative study revealed that CYPT enhanced reflective learning for tutors as well as tutees, which could not be fostered to the same degree by conventional faculty-led teaching. Taken together, we showed that CYPT could be beneficial for undergraduate medical interview training.

Although several systematic or narrative reviews suggested that the impact of near-peer teaching appeared to be at least equivalent to that of conventional faculty-led teaching, no studies have assessed the non-inferiority of near-peer tutoring [1, 4, 7, 11]. Our study demonstrated that the most recent OSCE scores of students taught using CYPT were not inferior to those of students taught using FLT.

The advantages and benefits of near-peer tutoring, regardless of the student tutors’ relative inexperience in teaching, have been much debated. These benefits include enhanced cognitive, psychomotor, and affective development of students and increased collegial behavior, attributable to the cognitive and social congruence among students [11, 22, 26].

The congruence concept in the context of near-peer tutoring is supported by Vygotsky’s theories of ‘scaffolded learning’ and ‘zone of proximal development’. The latter is defined as ‘The distance between the actual developmental level as determined by independent problem solving and the level of potential development as determined through problem solving under adult guidance or in collaboration with more capable peers.’ According to this theory, the learning level of the tutors is marginally higher than that of the tutees, enabling tutors to ‘scaffold’ the learning experiences of the tutees, thus helping to deepen their understanding of novel content [27, 28].

The qualitative findings of this study clarified the interaction between tutors and tutees in CYPT and demonstrated how the concepts related to cognitive and social congruence work in the context of undergraduate communication training. In the sessions, the tutees looked to the tutors as role models. Further, the tutors scaffolded the learning process for the tutees in the medical interview and communication sessions, on the assumption that tutees are generally unfamiliar with these topics [29]. In terms of ‘reflection on action,’ the tutors not only scaffolded the off-the-job training during the sessions, but also provided impetus for further self-learning among the tutees including their on-the-job training as medical students [30, 31]. The concept of a safe learning environment implies the presence of an underlying social congruence among tutees and tutors, while the concepts of effective feedback and practical advice presuppose cognitive congruence [32]. Interestingly, one of tutees anticipated that the tutors’ teaching experience would trigger self-reflection among the tutors [33]. Tutors reported benefiting professionally from their role and engaged in self-reflection as anticipated by the tutee. Furthermore, one of tutors reflected on his or her usual attitude as a learner when being taught by the faculty (Table 2). As a whole, from the perspective of reflective learning, CYPT could become a powerful tool triggering both reflection in action and reflection on action in medical students [32].

The literature discusses the rationale behind the implementation of CYPT. Some researchers report that financial issues or staff recruitment difficulties featured in their decision to establish a CYPT program [1, 3]. However, it is ethically difficult to defend implementing a CYPT program to replace staff with students despite resource and finance-related issues without any demonstrable educational advantage to the students. Our qualitative results revealed that working as a tutor could create a psychological burden on senior students, but that this conflict could be resolved with preliminary teacher training [34]. To manage student-centered CYPT effectively, the faculty could assign alternative responsibilities to the tutors to reduce their psychological burden, implement effective tutor training, and monitor and support the tutors closely [35]. Based on the findings of previous studies as well as our own, CYTP can be used in basic medical interview training for junior medical students as an adjunct to teaching by the faculty.

### Strengths and limitations

The strength of this paper is in its design. First, the main interest of CYPT research is the question of its efficacy in comparison with FLT. The trial designed for this study unequivocally demonstrated the non-inferiority of CYPT. Secondly, this mixed-methods study, combining the quantitative findings of non-inferiority and the qualitative results demonstrating the educational benefits of CYPT, effectively corroborated the comprehensive superiority of CYPT over FLT.

One limitation of our study was the relatively small amount of educational intervention. Our pre-clinical training program was an intensive course including four sessions of medical interviews. CYPT was adopted in only one of four communication training sessions and was conducted by only six student tutors. Although we understand that the intervention was on a smaller scale than that of previous studies and students were taught by faculty in other three sessions, we started this study with minimum intervention in view of the possible ethical problems that a more thorough-going intervention might have raised. Taking this limitation into account, incorporating CYPT into the communication training program should be started with a low amount of educational intervention. Another limitation was the teaching competency of the tutors. One hour of tutor-training might have been insufficient to achieve the desired outcomes. Further, we did not assess the tutors’ medical interview skills prior to their teaching session. While indeed this might have reduced the strength of the study, our chief concern was to decrease the burden on the tutors of preparing for their teaching sessions. The third limitation was the method of evaluating outcomes in this study. We assessed the students’ outcomes using the OSCE with a single encounter lasting ten minutes. Although it would have been ideal to assess the students after multiple encounters, this was not feasible in the setting of a government-run OSCE. The last potential limitation was the robustness of our qualitative results. Given the constraints of our study, we were able to hold only two focus groups. Although we have considered the issue of dependability of our qualitative data, we believe that we made the best effort possible to enhance the credibility, transferrability and confirmability of the qualitative results. Further studies are needed to overcome these limitations.

## Conclusions

CYPT can have an innovative and powerful impact on undergraduate communication training because the objective educational outcome of cross-year tutoring was not inferior to that of faculty-led training and was heuristic for both the tutees and tutors.

## Abbreviations

CATO: Common Achievement Test Organization
CI: Confidence Interval
CYPT: Cross-year peer tutoring
FLT: Faculty-led training
OSCE: Objective Structured Clinical Examination

## References

[1] Burgess A, McGregor D, Mellis C (2014). Medical students as peer tutors: a systematic review. BMC Med Educ..

[2] Ten Cate O, Durning S (2007). Peer teaching in medical education: twelve reasons to move from theory to practice. Med Teach.

[3] Ross MT, Cameron HS (2007). Peer assisted learning: a planning and implementation framework: AMEE guide no. 30. Med Teach..

[4] Santee J, Garavalia L (2006). Peer tutoring programs in health professions schools. Am J Pharm Educ.

[5] Shariq O, Alexopoulos AS, Razik F, Currie J, Salooja N (2013). Teaching skills training for medical students. Clin Teach.

[6] Soriano RP, Blatt B, Coplit L, CichoskiKelly E, Kosowicz L, Newman L, et al. Teaching medical students how to teach: a national survey of students-as-teachers programs in U.S. medical schools. Acad Med. 2010;85(11):1725–31. 10.1097/ACM.0b013e3181f53273 20881824

[7] Yu TC, Wilson NC, Singh PP, Lemanu DP, Hawken SJ, Hill AG (2011). Medical students-as-teachers: a systematic review of peer-assisted teaching during medical school. Adv Med Educ Pract..

[8] Preece R, Dickinson EC, Sherif M, Ibrahim Y, Ninan AS, Aildasani L, et al. Peer-assisted teaching of basic surgical skills. Med Educ Online. 2015;20:27579. 10.3402/meo.v20.27579 PMC4456402 26044400

[9] Charlier N, Van Der Stock L, Iserbyt P (2016). Peer-assisted learning in cardiopulmonary resuscitation: the jigsaw model. J Emerg Med.

[10] Silbert BI, Lake FR (2012). Peer-assisted learning in teaching clinical examination to junior medical students. Med Teach..

[11] Bene KL, Bergus G (2014). When learners become teachers: a review of peer teaching in medical student education. Fam Med.

[12] Nestel D, Kidd J (2005). Peer assisted learning in patient-centred interviewing: the impact on student tutors. Med Teach..

[13] Nestel D, Kidd J (2003). Peer tutoring in patient-centred interviewing skills: experience of a project for first-year students. Med Teach..

[14] Steele DJ, Medder JD, Turner P (2000). A comparison of learning outcomes and attitudes in student- versus faculty-led problem-based learning: an experimental study. Med Educ.

[15] Kassab S, Abu-Hijleh MF, Al-Shboul Q, Hamdy H (2005). Student-led tutorials in problem-based learning: educational outcomes and students’ perceptions. Med Teach..

[16] Heckmann JG, Dutsch M, Rauch C, Lang C, Weih M, Schwab S (2008). Effects of peer-assisted training during the neurology clerkship: a randomized controlled study. Eur J Neurol.

[17] Tolsgaard MG, Ringsted C (2014). Using equivalence designs to improve methodological rigor in medical education trials. Med Educ.

[18] Tolsgaard MG, Kulasegaram KM, Ringsted C (2017). Practical trials in medical education: linking theory, practice and decision making. Med Educ.

[19] Weeks DL, Molsberry DM (2008). Pediatric advanced life support re-training by videoconferencing compared to face-to-face instruction: a planned non-inferiority trial. Resuscitation.

[20] Taylor R, Jung J, Loewen P, Spencer C, Dossa A, de Lemos J (2013). Online versus live delivery of education to pharmacists in a large multicentre health region: a non-inferiority assessment of learning outcomes. Can J Hosp Pharm.

[21] Piaggio G, Elbourne DR, Pocock SJ, Evans SJ, Altman DG, Group C (2012). Reporting of noninferiority and equivalence randomized trials: extension of the CONSORT 2010 statement. JAMA.

[22] Ten Cate O, Durning S (2007). Dimensions and psychology of peer teaching in medical education. Med Teach..

[23] Buckley S, Zamora J (2007). Effects of participation in a cross year peer tutoring programme in clinical examination skills on volunteer tutors’ skills and attitudes towards teachers and teaching. BMC Med Educ.

[24] Marton GE, McCullough B, Ramnanan CJ (2015). A review of teaching skills development programmes for medical students. Med Educ.

[25] Kinoshita Y (2007). Modified grounded theory approach.

[26] Blohm M, Lauter J, Branchereau S, Krautter M, Kohl-Hackert N, Junger J, et al. “peer-assisted learning” (PAL) in the skills-lab--an inventory at the medical faculties of the Federal Republic of Germany. GMS Z Med Ausbild. 2015;32(1):Doc10. 10.3205/zma000952 PMC4330629 25699102

[27] Topping KJ (1996). The effectiveness of peer tutoring in further and higher education: a typology and review of the literature. High Educ.

[28] Vygotsky LS. Mind in society: The development of higher psychological processes. Cambridge: Harvard University Press; 1980. http://www.hup.harvard.edu/about/.

[29] Berghmans I, Druine N, Dochy F, Struyven K (2012). A facilitative versus directive approach in training clinical skills? Investigating students’ clinical performance and perceptions. Perspect Med Educ.

[30] Srivastava TK, Waghmare LS, Mishra VP, Rawekar AT, Quazi N, Jagzape AT (2015). Peer teaching to Foster learning in physiology. J Clin Diagn Res.

[31] Schon DA, DeSanctis V (1986). The reflective practitioner: how professionals think in action. J Cont High Educ.

[32] Hum L, Maccaro J, Park SE (2014). Cross-year Peer Tutoring in Healthcare and Dental Education: A Review of the Literature. J Curric Teach.

[33] Menezes A, Burgess A, Clarke AJ, Mellis C (2016). Peer-assisted learning in medical school: tutees’ perspective. Adv Med Educ Pract.

[34] Burgess A, Clark T, Chapman R, Mellis C (2013). Senior medical students as peer examiners in an OSCE. Med Teach..

[35] Erlich DR, Shaughnessy AF (2014). Student-teacher education programme (STEP) by step: transforming medical students into competent, confident teachers. Med Teach.

