# Correction to: medical students can teach communication skills – a mixed methods study of cross-year peer tutoring

**DOI:** 10.1186/s12909-017-1014-0

**Published:** 2017-10-20

**Authors:** Osamu Nomura, Hirotaka Onishi, Hiroyuki Kato

**Affiliations:** 1 Department of Integrated Medical Education, Graduate School of Medicine, Hirosaki University, 1 Zaifu-cho, Hirosaki city, Aomori Japan; 2 Division of Pediatric Emergency Medicine, Tokyo Metropolitan Children’s Hospital, 2-8-29 Musashidai, Fuchu city, Tokyo Japan; 3 International Research Center for Medical Education, Graduate School of Medicine, The University of Tokyo, 7-3-1 Hongo, Bunkyo-ku, Tokyo, Japan

## Correction

Following publication of the original article [1], the authors reported that the word “Tutee” on the right-hand side of Fig. 3 should read “Tutor”.

**Fig. 3:**
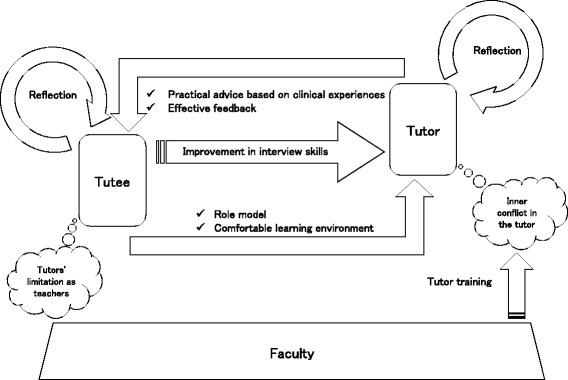
ᅟ
